# Tocolytic activity of the hydroalcoholic extract of *Peperomia galioides* Kunth (congona) leaves in non-pregnant rat uterus

**DOI:** 10.17843/rpmesp.2026.431.15504

**Published:** 2026-03-31

**Authors:** Withney Quinto-Aylas, Luz Poma-Aquino, Daisy Flores-Cortez, Eduardo Villalobos-Pacheco, Cecilia Ignacio-Punin

**Affiliations:** 1 Pharmacology Laboratory, Faculty of Human Medicine, Universidad Nacional Mayor de San Marcos, Lima, Peru.; 2 Research Group in Basic and Clinical Pharmacology of Drugs and Natural Products (FARMANAT), Lima, Perú.

**Keywords:** Plant Extracts, Peperomia, Uterine Contraction, Tocolytic Agents

## Abstract

**Objective.:**

To evaluate the effect of the hydroalcoholic extract of *Peperomia galioides* Kunth (congona) leaves on uterine contractility in an isolated non-pregnant rat uterus model.

**Materials and methods.:**

An *in vitro* experimental study was conducted with 15 female rats pretreated with estrogen 48 hours prior to the experiment. Uterine horns were sectioned into 10 mm segments and mounted in an isolated organ bath under a basal tension of 1 g. The tissue was stimulated with oxytocin (10-⁶ M) and subsequently exposed to the hydroalcoholic extract of *P. galioides* (0.1%, 1%, and 10%) and the control. The frequency, duration, and intensity of uterine contractions were evaluated, and the percentage of inhibition was calculated. Additionally, a preliminary phytochemical screening of the extract was performed.

**Results.:**

All evaluated concentrations of *P. galioides* significantly reduced the frequency of oxytocin-induced uterine contractions compared to basal values and the control group (p < 0.001). Regarding intensity and duration, only the 1% and 10% concentrations produced a significant decrease relative to basal values (p < 0.05). However, only the 10% extract significantly inhibited intensity and duration compared to the control group. Phytochemical analysis revealed a high content of phenolic compounds and flavonoids.

**Conclusion.:**

The hydroalcoholic extract of *P. galioides* Kunth leaves exerts an inhibitory effect on uterine contractility in the isolated rat uterus, supporting its potential tocolytic action.

## INTRODUCTION

Preterm birth remains one of the primary challenges in public health due to its considerable impact on perinatal morbidity and mortality. It is estimated to represent approximately 70% of neonatal deaths and 36% of infant deaths globally ^1^^,^^2^. According to the World Health Organization (WHO), around 15 million infants are born prematurely each year, with more than one million neonatal deaths attributed to complications associated with prematurity ^3^^,^^4^.

Tocolytic agents, whose objective is to delay labor to allow for fetal maturation, have shown limited efficacy in significantly prolonging gestation. Furthermore, many of these drugs are associated with maternal and fetal adverse effects—such as tachycardia, hypotension, arrhythmias, premature closure of the ductus arteriosus, and systemic toxicity—that restrict their clinical use. These limitations, along with the high cost of some medications and restricted access in low-resource contexts, highlight the urgent need for safer and more effective therapeutic alternatives ^2^^,^^5^.

It is estimated that approximately 80% of the world population resorts to the use of medicinal plants to satisfy basic health care needs ^6^. Various studies have demonstrated uterorelaxant (utero-inhibitory) effects in plants such as *Mentha pulegium*, *Justicia flava*, *Ananas comosus*, *Pimpinella anisum*, *Bryophyllum pinnatum*, and *Cinnamomum verum* ^(7-10)^. In the Peruvian context, the empirical use of plant species such as celery, oregano, rue, basil, chamomile, and golden berry during labor is common ^11^^,^^12^.

*Peperomia galioides* Kunth, popularly known as "congona," is a plant native to South America, especially the Andean regions of Peru, Ecuador, Bolivia, and Colombia. Traditionally, the leaves and stems of plants from the *Peperomia* genus are used in the form of infusions or decoctions for the management of gastrointestinal spasmodic pain, menstrual cramps, and other associated painful clinical presentations ^(13-15)^. A study on the use of medicinal plants during labor in an Andean region of Peru reported that 10.2% of pregnant women consumed congona during this stage ^16^. Other research also documents its intake in the form of infusions during labor, primarily for oxytocic purposes (47.8%) ^17^.

Although it is empirically considered to provide benefits during labor and the postpartum period, the scientific evidence regarding its pharmacological effects is limited and inconsistent. Preliminary experimental studies have reported sedative and anti-inflammatory properties, possibly mediated by the inhibition of cyclooxygenase-2 and other inflammatory mediators, suggesting a potential tocolytic effect not yet confirmed ^18^^,^^19^.

Given its traditional use during labor and the absence of studies evaluating its efficacy as a tocolytic agent, a scientific investigation of this plant is justified. The present study aimed to evaluate the effect of the hydroalcoholic extract of *P. galioides* leaves on uterine contractility in an experimental model of isolated non-pregnant rat uterus. It is expected that the results will provide preliminary evidence to support its rational use in pregnant populations and establish the basis for future research oriented towards elucidating its mechanism of action, toxicity profile, and therapeutic potential.

**KEY MESSAGES**

Motivation for the study. *Peperomia galioides* Kunth is a plant native to the Andean regions of South America, traditionally used for its antispasmodic properties during labor; however, the scientific evidence supporting this ethnomedicinal use is limited.

Main findings. The present study demonstrated that the leaf extract of *P. galioides* exerts an inhibitory effect on uterine contractility in non-pregnant rats. Phytochemical analysis showed a high content of phenolic compounds and flavonoids, which could partially contribute to this activity.

Public health implications. These findings suggest that *P. galioides* could constitute a potential natural tocolytic agent in the management of threatened preterm labor; however, additional clinical and safety studies are required to validate its therapeutic potential.

## MATERIALS AND METHODS

### Study design

An *in vitro* experimental study was conducted using an isolated organ model. The experimental unit corresponded to each segment of rat uterine horn mounted in the organ bath system. Fifteen adult female rats were used, from which two uterine horns were obtained per animal. Each horn was sectioned into two segments of approximately 10 mm, obtaining a total of four uterine segments per animal.

The uterine segments were randomly assigned to the different experimental treatments using simple random sampling. Each uterine segment was identified with a code and recorded in a Microsoft Excel spreadsheet. Subsequently, the "RANDBETWEEN" function was used to generate the randomization sequence and assign the uterine segments to the different experimental groups. The evaluated groups included a control and three groups treated with hydroalcoholic extract of *P. galioides* at concentrations of 0.1%, 1%, and 10%. Each tissue preparation was used only once during the experimental protocol.

The sample size was established based on previous pharmacological studies conducted in isolated rat uterus models evaluating oxytocin-induced myometrial contractility, in which between 10 and 15 tissue preparations per experimental group are used to detect significant changes in uterine contraction parameters ^20^^,^^21^. Considering the variability reported for this model and the expected magnitude of effect for compounds with spasmolytic activity, a minimum size of 15 uterine segments per experimental group was established, which is considered adequate to identify statistically significant differences under controlled experimental conditions.

### Chemical substances and drugs

All salts used for the preparation of the Tyrode solution and for phytochemical screening were of analytical grade and purchased from Sigma Chemical (St. Louis, MO, USA). Oxytocin (Pitocin 10 U/mL®, SIEGFRIED Laboratories, Lima, Peru) and estradiol cypionate (Estrogal 2 mg/mL®) were obtained from a local pharmacy.

### Collection of plant material and extract preparation

*P. galioides* Kunth (commonly known as congona) was collected in the town of Chichipe, Huayllapampa district (Ancash, Peru), located at 3132 m a.s.l. The plants were selected ensuring the absence of physical damage, exposure to pesticides, or other contaminants. Taxonomic identification was performed by a certified biologist from the Natural History Museum of the Universidad Nacional Mayor de San Marcos (Certificate 147-USM-MHN-2024).

The leaves were washed with distilled water, dried for seven days at 40 °C with continuous ventilation, and ground into a uniform powder. The hydroalcoholic extract was prepared by macerating 250 g of powdered leaves in 1 L of 70% ethanol for 7 days with daily agitation. The extract was filtered with Whatman No. 3 paper, and the filtrate was evaporated at 40 °C using a rotary evaporator (Buchi®). The concentrated extract was subsequently dried at 40 °C for 7 days and stored at −30 °C.

The extraction yield was 2%, equivalent to 2 g of crude hydroalcoholic extract for every 100 g of macerated plant material. For bioactivity assays, the extracts were dissolved in 10% Tween 80.

### Preliminary phytochemical screening

The hydroalcoholic extract of *P. galioides* was subjected to qualitative screening for secondary metabolites using previously described standard phytochemical methods ^22^. The tests included: Liebermann-Burchard reaction for sterols and triterpenoids; Shinoda test for flavonoids; ferric chloride test for phenolic compounds; and Dragendorff, Wagner, and Mayer tests for alkaloids.

Additionally, the gelatin test for tannins, Baljet reaction for saponins, Bortanger reaction for anthraquinones and naphthoquinones, Salkowski reaction for terpenoids and steroidal flavonoids, Bertrand test for cardiotonic glycosides and sesquiterpene lactones, and the vanillin/H₂SO₄ assay to determine the foam index were performed. All reactions were carried out under controlled laboratory conditions, using analytical grade reagents and standardized qualitative methodologies to ensure reproducibility.

### Experimental animals

Fifteen virgin adult female Holtzman rats, three months old and weighing between 250-300 g, were used, obtained from the bioterium of the Universidad Nacional Agraria La Molina (Lima, Peru). The animals were housed in the bioterium of the Faculty of Medicine of the Universidad Nacional Mayor de San Marcos (UNMSM) under controlled conditions of temperature (21-25 °C), humidity (50-60%), and a 12-hour light/dark cycle, with free access to food and water. An acclimatization period of seven days was conducted before the experiment.

The inclusion criteria, defined *a priori*, included healthy adult female rats, virgins, within the established weight range. Animals presenting clinical signs of disease, lesions, significant weight loss, or any condition that could affect the physiological response of the uterine tissue were excluded. Likewise, uterine segments that did not present adequate basal contractile activity or did not respond to the oxytocin stimulus during the stabilization period were discarded.

During the course of the experiment, no animals or experimental units were excluded from the analysis. All uterine segments obtained met the established viability criteria, showing basal contractile activity and adequate response to the oxytocin stimulus during the stabilization period. Therefore, all data obtained were included in the final analysis.

### Preparation and mounting of the isolated uterus

The rats were pretreated by intramuscular injection of 0.6 mg of estradiol cypionate (Estrogal® 2 mg/mL) 48 hours before the experiment. The animals were anesthetized with sodium pentobarbital (60 mg/kg, i.p.) and sacrificed by cervical dislocation.

A midline laparotomy was performed to extract the uterus, which was cleaned of connective tissue and placed in a Petri dish with Tyrode solution at 37 °C. Each uterine horn was sectioned into 10 mm fragments and mounted in a 5 mL organ bath with oxygenated Tyrode solution (95% O₂, 5% CO₂) maintained at 37 °C.

The Tyrode solution had the following composition (per liter): NaCl 136 mM, KCl 2.68 mM, CaCl₂ 1.80 mM, MgCl₂ 1.05 mM, NaHCO₃ 11.9 mM, NaH₂PO₄ 0.42 mM, and glucose 5.55 mM. The tissue strips were fixed with surgical thread and connected to an isometric force transducer (ADInstruments, Australia), allowing the recording of contractile activity through LabChart® software.

The preparations were stabilized for 60 minutes, with solution changes every 20 minutes, maintaining a basal tension of 1 g. To minimize possible confounding variables and ensure the reproducibility of uterine contractility measurements, all experiments were conducted under standardized experimental conditions of temperature, oxygenation, and basal tension in the organ bath system.

### Pharmacological assays

After the stabilization period, basal contractile activity was recorded for three minutes. Subsequently, the uterine tissue was stimulated with 0.2 mL of oxytocin solution (10⁻⁶ M), and the contraction was recorded for another three minutes. Then, the hydroalcoholic extracts (0.1%, 1%, and 10%) were administered sequentially at three-minute intervals.

At the end of the experiment, five consecutive washes were performed, and the recovery of oxytocin-induced activity was evaluated to confirm tissue viability. The parameters analyzed were frequency (contractions/3 minutes), intensity (g of force), and duration (seconds) of the uterine contractions.

Due to the nature of the experiment and the direct handling of the preparations in the organ bath system, the researcher who performed the recordings of the contractile activity was aware of the treatments administered. However, the measurements of uterine contractility parameters (frequency, duration, and intensity) were obtained through an automated isometric recording system and subsequently analyzed using specialized software, which reduced the possibility of bias in the evaluation of the results and data analysis.

### Statistical analysis

Data normality was evaluated using the Shapiro-Wilk test, which indicated a non-normal distribution (p < 0.05). Consequently, the Wilcoxon signed-rank test was used to compare values before and after treatment, and the Kruskal-Wallis test was used for comparisons between groups. For all analyses, the IBM SPSS version 26 statistical package was used. A p-value < 0.05 was accepted as significant.

The percentage of inhibition for each parameter was calculated using the following formula:


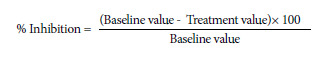


### Ethical aspects

The study was approved by the Ethics Committee of the Faculty of Medicine of the UNMSM (code: 0072-2024), which verified the suitability of the project and guaranteed that adequate care was provided during housing, environmental factors, restraint practices, injection, and euthanasia of the rats. All procedures were performed in accordance with the guidelines for the care and use of laboratory animals and international standards for preclinical research, ensuring proper handling, restraint, anesthesia, and euthanasia ^23^^,^^24^. Furthermore, the study was developed in compliance with the guidelines established by the ARRIVE declaration ^25^.

## RESULTS

### Qualitative phytochemical profile of the hydroalcoholic extract of *Peperomia galioides* Kunth

The qualitative phytochemical screening of the hydroalcoholic extract of *P. galioides* (congona) revealed the presence of various secondary metabolites. A high presence (+++) of phenolic compounds was observed through the ferric chloride test, as well as glycosides detected by the vanillin/H₂SO₄ test. The Shinoda test also showed a high presence (+++) of flavonoids.

On the other hand, a moderate presence (++) of cardiotonic glycosides and sesquiterpene lactones was detected through the Baljet test. A low presence (+) of anthraquinones and naphthoquinones was observed through the Bortanger test, as well as terpenoids and flavonoids with a steroid nucleus through the Salkowski test.

No evidence (−) was found for tannins (gelatin test), steroids and triterpenoids (Liebermann-Burchard test), alkaloids (Dragendorff, Wagner, Bertrand, and Mayer tests), or saponins (foam index).

### Effect of the hydroalcoholic extract of *Peperomia galioides* Kunth on oxytocin-induced uterine contraction parameters

**Figure 1:**
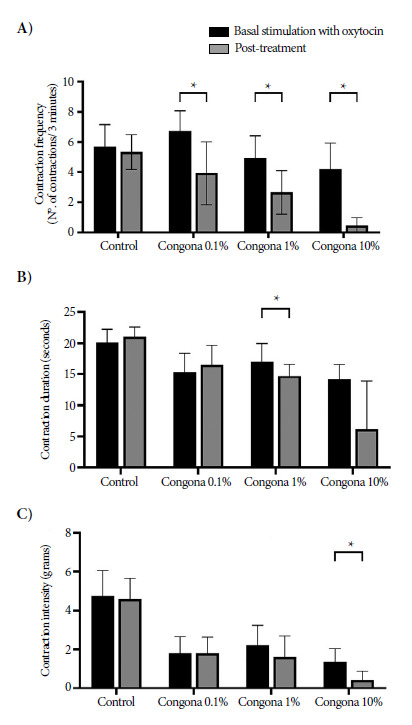
Effect of the hydroalcoholic extract of *Peperomia galioides* Kunth on the frequency, duration, and intensity of oxytocin-induced uterine contractions in rats. The rats were treated with different concentrations of the extract (0.1%, 1%, and 10%) and compared with the control group (oxytocin alone). Three parameters were evaluated: contraction frequency (A), duration (B), and intensity (C). Data are expressed as mean ± standard deviation.

The hydroalcoholic extract of *P. galioides* exerted modulating effects on oxytocin-induced uterine contractility, evaluated through the frequency, duration, and intensity of contractions (Figure 1).

Statistical analysis was performed using the non-parametric Wilcoxon signed-rank test for paired samples, adjusted by the Benjamini, Krieger, and Yekutieli method (FDR 1%). p < 0.05 (*).

In relation to contraction frequency, all evaluated extract concentrations significantly reduced oxytocin-induced contractions compared to the control group (p < 0.05).

Regarding contraction duration, only the 1% and 10% concentrations produced a statistically significant reduction compared to the 0.1% concentration and the control group (p < 0.05). No significant differences were detected between the 0.1% and 1% concentrations, nor in comparison with the control.

As for contraction intensity, a statistically significant reduction was also observed with the 1% and 10% concentrations compared to the group treated with 0.1% and the control group (p < 0.05).

Altogether, these findings suggest a concentration-dependent effect, in which the 1% and 10% concentrations exerted a significant inhibitory effect on all contractile parameters in the isolated rat uterus model.

### Inhibition percentage of uterine contraction parameters

**Figure 2:**
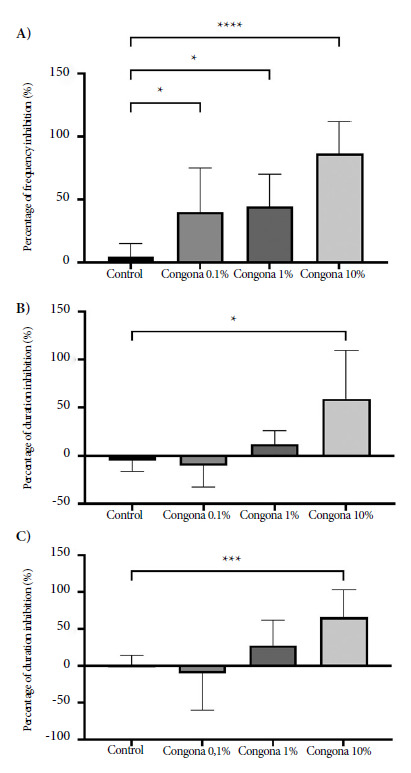
Percentage of inhibition of the hydroalcoholic extract of *Peperomia galioides* Kunth on oxytocin-induced uterine contraction parameters. The percentage of inhibition relative to the control group was calculated for the three parameters evaluated: frequency (A), duration (B), and intensity (C) of the uterine contraction. The extract was evaluated at concentrations of 0.1%, 1%, and 10%. Data are presented as mean ± standard deviation.

Figure 2 shows the percentage of inhibition relative to oxytocin-induced contractions for each uterine contractility parameter.

Statistical analysis was performed using the non-parametric Kruskal-Wallis test, with 1% FDR correction. p < 0.05 (*), p < 0.01 (**), p < 0.001 (***).

The 10% extract presented the highest percentage of inhibition in the three parameters evaluated. For contraction frequency, the 0.1%, 1% (p < 0.05), and 10% (p < 0.001) concentrations differed significantly from the control group.

Regarding contraction duration, only the 10% concentration produced a significantly higher inhibition compared to the control group (p < 0.05), as well as with the 0.1% and 1% concentrations. No significant differences were observed between the 0.1% and 1% concentrations.

With respect to contraction intensity, only the 10% extract showed a significant difference against the control (p < 0.001), while the 0.1% and 1% concentrations did not differ significantly from the control or from each other.

## DISCUSSION

The results demonstrated that the extract of *P. galioides* Kunth inhibits the three evaluated parameters of uterine contraction in a dose-dependent manner. In particular, the contraction frequency was significantly lower than that of the control group in all evaluated concentrations, with the highest concentration being the most effective (p < 0.05). Likewise, when analyzing the intensity inhibition percentage, only the 10% concentration showed a statistically significant difference, indicating that at high doses, the extract possesses a clear ability to attenuate the myometrial contractile force induced by oxytocin.

Regarding the duration and intensity of contractions, although a decreasing trend was observed in absolute values as the extract concentration increased, the analysis of the inhibition percentage indicated that only the 10% concentration significantly reduced the contraction intensity compared to the control (p < 0.01). This effect supports the hypothesis that *P. galioides* could modulate myometrial contractility by interfering with the physiological mechanisms that regulate uterine contraction, particularly by reducing the duration of effective contractile events. It has been described that uterine smooth muscle contractility depends largely on calcium entry through voltage-dependent channels and on the intracellular mechanisms that regulate the generation of myometrial action potentials; therefore, the inhibition of these pathways can decrease the force and duration of the contraction ^26^^,^^27^.

The phytochemical screening of the hydroalcoholic extract of *P. galioides* Kunth (congona) revealed the presence of phenolic compounds, flavonoids, and glycosides, along with traces of other secondary metabolites. From a pharmacological standpoint, these findings are relevant, as both flavonoids and phenolic compounds have been widely associated with anti-inflammatory, antioxidant, and spasmolytic activities previously described in species of the *Peperomia* genus ^28^.

The identification of flavonoids with high evidence (+++) agrees with previous studies highlighting their role in the modulation of smooth muscle contractility and the inhibition of pro-inflammatory mediators, helping to explain the potential tocolytic or antispasmodic effects traditionally attributed to the plant. Another relevant aspect is the estrogenic activity described in other *Peperomia* species, which could represent an additional mechanism. It has been reported that phenolic compounds present in this genus have an affinity for estrogen receptors, suggesting a potential to modulate hormonal pathways related to uterine contractility ^29^.

Some bioactive compounds isolated from *Peperomia pellucida* (such as derivatives of dillapiol and pellucidin A) have demonstrated modulating effects on estrogen receptors ^29^. In molecular docking studies, estrogenic activity appeared to be mediated by a classic ligand-dependent mechanism, as suggested by the binding interactions between these compounds and estrogen receptors.

Although other metabolites, such as cardiotonic glycosides and sesquiterpene lactones, were identified with lower intensity, their potential pharmacological contribution cannot be ruled out and requires additional studies to evaluate their specific role.

Furthermore, previous studies have demonstrated that pellucidin A is capable of inhibiting cyclooxygenase-2 (COX-2) and inducible nitric oxide synthase (iNOS), which could mediate a relaxing effect by reducing the synthesis of pro-contractile prostaglandins and nitric oxide in smooth muscle cells ^30^^,^^31^. This mechanism is shared with first-line clinical tocolytics, such as NSAIDs, suggesting a plausible rational basis for its potential therapeutic use ^5^.

*P. pellucida* also presents vasorelaxant effects mediated by nitric oxide-dependent mechanisms ^32^. These effects have been observed in vascular tissues such as the aorta, where relaxation is evident against contractile stimuli like phenylephrine and KCl. Given that uterine smooth muscle shares physiological similarities with other types of smooth muscle, it is possible that *P. pellucida* also exerts a relaxing effect at the uterine level, particularly in contexts where the regulation of muscle tone is essential to maintain uterine quiescence and control labor. Although nitric oxide donors like nitroglycerin are not routinely used as tocolytics, they may be used in specific clinical contexts due to their relaxing effect on uterine smooth muscle ^33^.

The activation of estrogen receptors increases endothelial production of nitric oxide, which plays a key role in uterine vasodilation during pregnancy ^34^. Since estrogen favors nitric oxide production, it could also have a central role in regulating the pattern and frequency of uterine contractions during labor. Therefore, it is plausible that the estrogenic activity of *P. pellucida* could modify contractile activity.

Despite the findings obtained, it is important to recognize some limitations of the study. In the first place, comparative trials with reference tocolytics were not performed, which limits the possibility of establishing direct comparisons on the potency or the pharmacological mechanism of the evaluated extract; however, this limitation does not invalidate the results, since the primary objective was to determine the modulating effect of the extract on uterine contractility in an experimental model of oxytocin-induced isolated uterus, widely used and validated to evaluate uterine activity. Likewise, the use of a crude hydroalcoholic extract constitutes another limitation, because it does not allow for the identification or quantification of the bioactive compounds responsible for the observed effect, which could generate variability in the chemical composition; nevertheless, this possible effect was minimized through the standardization of the collection, identification, drying, and extraction process of the plant material, as well as through the performance of a preliminary phytochemical screening that confirmed the presence of potentially active secondary metabolites.

In general terms, these results partially support the traditional use of congona as a medicinal plant and provide a basis for future research oriented toward the characterization and quantification of its bioactive compounds, as well as the performance of comparative trials to clarify the mechanism of action with greater precision. In this context, future research should prioritize specific *in vitro* and *in vivo* assays in uterine tissue to define the pharmacodynamic profile of this plant and evaluate its potential obstetric application in preterm labor. Furthermore, considering that the findings come from an experimental model in isolated rat uterus, their extrapolation to other species, including humans, must be done with caution. Therefore, additional studies evaluating its efficacy and safety are required before considering any possible clinical application.

In conclusion, the results of the present study demonstrate that the hydroalcoholic extract of *Peperomia galioides* Kunth (congona) leaves exerts a significant inhibitory effect on oxytocin-induced uterine contractility in an isolated non-pregnant rat uterus model, particularly at the 10% concentration.
