# Supplementary material for: Tocolytic activity of the hydroalcoholic extract of Peperomia galioides Kunth (congona) leaves in non-pregnant rat uterus
Source: Rev Peru Med Exp Salud Publica. 2026 Mar 31;43(1):61–8. doi: 10.17843/rpmesp.2026.431.15504 (PMC13245984; doi:10.17843/rpmesp.2026.431.15504)
Supplement: Translation

# Translation: Actividad tocolítica del extracto hidroalcohólico de hojas de *Peperomia galioides* Kunth (congona) en útero de ratas no gestantes

**Authors:** Withney Quinto-Aylas, Luz Poma-Aquino, Daisy Flores-Cortez, Eduardo Villalobos-Pacheco, Cecilia Ignacio-Punin

**Affiliations:** 1 Laboratorio de Farmacología, Facultad de Medicina Humana, Universidad Nacional Mayor de San Marcos, Lima, Perú.; 2 Research Group in Basic and Clinical Pharmacology of Drugs and Natural Products (FARMANAT), Lima, Peru.

**Keywords:** Extractos de Plantas, Peperomia, Contracción Uterina, Tocolítico

## Abstract

**Objetivo.:**

Evaluar el efecto del extracto hidroalcohólico de hojas de *Peperomia galioides* Kunth (congona) sobre la contractilidad uterina en un modelo de útero aislado de rata no gestante.

**Materiales y métodos.:**

Se realizó un estudio experimental *in vitro* con 15 ratas hembra pretratadas con estrógeno 48 horas previo al experimento. Los cuernos uterinos fueron seccionados en segmentos de 10 mm y montados en un baño de órgano aislado bajo una tensión basal de 1 g. El tejido fue estimulado con oxitocina (10^-^⁶ M) y posteriormente expuesto a extracto hidroalcohólico de *P. galioides* (0,1%, 1% y 10%), y al control. Se evaluó la frecuencia, duración e intensidad de las contracciones uterinas y se calculó el porcentaje de inhibición. Asimismo, se realizó un tamizaje fitoquímico preliminar del extracto.

**Resultados.:**

Todas las concentraciones evaluadas de *P. galioides* redujeron significativamente la frecuencia de las contracciones uterinas inducidas por oxitocina en comparación con los valores basales y el grupo control (p < 0,001). En cuanto a la intensidad y duración, solo las concentraciones al 1% y 10% produjeron una disminución significativa respecto a los valores basales (p < 0,05). Sin embargo, únicamente el extracto al 10% inhibió significativamente la intensidad y duración, en comparación con el grupo control. El análisis fitoquímico reveló un alto contenido de compuestos fenólicos y flavonoides.

**Conclusión.:**

El extracto hidroalcohólico de hojas de *P. galioides* Kunth ejerce un efecto inhibidor sobre la contractilidad uterina en útero aislado de rata, lo que respalda su potencial acción tocolítica.

## INTRODUCCIÓN

El parto prematuro continúa siendo uno de los principales desafíos en la salud pública debido a su considerable impacto en la morbilidad y mortalidad perinatal. Se estima que representa aproximadamente el 70% de las muertes neonatales y el 36% de las muertes infantiles a nivel mundial ^1^^,^^2^. Según la Organización Mundial de la Salud (OMS), alrededor de 15 millones de lactantes nacen prematuramente cada año, con más de un millón de muertes neonatales atribuidas a complicaciones asociadas a la prematuridad ^3^^,^^4^.

Los agentes tocolíticos, cuyo objetivo es retrasar el parto para permitir la maduración fetal, han demostrado una eficacia limitada para prolongar significativamente la gestación. Además, muchos de estos fármacos se asocian con efectos adversos maternos y fetales -como taquicardia, hipotensión, arritmias, cierre prematuro del conducto arterioso y toxicidad sistémica- que restringen su uso clínico. Estas limitaciones, junto con el alto costo de algunos medicamentos y el acceso restringido en contextos de bajos recursos, evidencian la necesidad urgente de alternativas terapéuticas más seguras y eficaces ^2^^,^^5^.

Se estima que aproximadamente el 80% de la población mundial recurre al uso de plantas medicinales para satisfacer necesidades básicas de atención en salud ^6^. Diversos estudios han demostrado efectos uterorrelajantes (uteroinhibidores) en plantas como *Mentha pulegium*, *Justicia flava, Ananas comosus, Pimpinella anisum, Bryophyllum pinnatum* y *Cinnamomum verum*^7^^-^^10^. En el contexto peruano, es común el uso empírico durante el trabajo de parto de especies vegetales como apio, orégano, ruda, albahaca, manzanilla y aguaymanto ^11^^,^^12^.

*Peperomia galioides* Kunth, conocida popularmente como “congona”, es una planta nativa de Sudamérica, especialmente de las regiones andinas de Perú, Ecuador, Bolivia y Colombia. Tradicionalmente, las hojas y tallos de plantas del género *Peperomia* se emplean en forma de infusiones o decocciones para el manejo de dolor espasmódico gastrointestinal, cólicos menstruales y otros cuadros dolorosos asociados ^13^^-^^15^. Un estudio sobre el uso de plantas medicinales durante el trabajo de parto en una región andina del Perú reportó que el 10,2% de las gestantes consumían congona en esta etapa ^(^^16^. Otras investigaciones también documentan su ingesta en forma de infusiones durante el trabajo de parto, principalmente con fines oxitócicos (47,8%) ^17^.

Aunque empíricamente se considera que proporciona beneficios durante el trabajo de parto y el periodo posparto, la evidencia científica sobre sus efectos farmacológicos es limitada e inconsistente. Estudios experimentales preliminares han reportado propiedades sedantes y antiinflamatorias, posiblemente mediadas por la inhibición de la ciclooxigenasa-2 y otros mediadores inflamatorios, lo que sugiere un potencial efecto tocolítico aún no confirmado ^18^^,^^19^.

Dado su uso tradicional durante el trabajo de parto y la ausencia de estudios que evalúen su eficacia como agente tocolítico, se justifica una investigación científica de esta planta. El presente estudio tuvo como objetivo evaluar el efecto del extracto hidroalcohólico de hojas de *P. galioides* sobre la contractilidad uterina en un modelo experimental de útero aislado de rata no gestante. Se espera que los resultados proporcionen evidencia preliminar para respaldar su uso racional en poblaciones gestantes y establecer las bases para futuras investigaciones orientadas a dilucidar su mecanismo de acción, perfil de toxicidad y potencial terapéutico.

**MENSAJE CLAVE**

Motivación para realizar el estudio. *Peperomia galioides* Kunth es una planta nativa de las regiones andinas de Sudamérica, tradicionalmente empleada por sus propiedades antiespasmódicas durante el trabajo de parto; sin embargo, la evidencia científica que respalda este uso etnomedicinal es limitada.

Principales hallazgos. El presente estudio demostró que el extracto de hojas de *P. galioides* ejerce un efecto inhibidor sobre la contractilidad uterina en ratas no gestantes. El análisis fitoquímico evidenció un alto contenido de compuestos fenólicos y flavonoides, los cuales podrían contribuir parcialmente a esta actividad.

Implicancias en salud pública. Estos hallazgos sugieren que *P. galioides* podría constituir un potencial agente tocolítico de origen natural en el manejo de la amenaza de parto prematuro; sin embargo, se requieren estudios adicionales en modelos clínicos y de seguridad para validar su potencial terapéutico.

## MATERIALES Y MÉTODOS

### Diseño del estudio

Se realizó un estudio experimental *in vitro* utilizando un modelo de órgano aislado. La unidad experimental correspondió a cada segmento de cuerno uterino de rata montado en el sistema de baño de órganos. Se emplearon 15 ratas hembra adultas, de las cuales se obtuvieron dos cuernos uterinos por animal. Cada cuerno fue seccionado en dos segmentos de aproximadamente 10 mm, obteniéndose un total de cuatro segmentos uterinos por animal.

Los segmentos uterinos fueron asignados aleatoriamente a los diferentes tratamientos experimentales mediante muestreo aleatorio simple. Cada segmento uterino fue identificado con un código y registrado en una hoja de cálculo de Microsoft Excel. Posteriormente, se utilizó la función “aleatorio.entre” para generar la secuencia de aleatorización y asignar los segmentos uterinos a los diferentes grupos experimentales. Los grupos evaluados incluyeron un control y tres grupos tratados con extracto hidroalcohólico de *P. galioides* a concentraciones de 0,1%, 1% y 10%. Cada preparación tisular fue utilizada una sola vez durante el protocolo experimental.

El tamaño muestral se estableció con base en estudios farmacológicos previos realizados en modelos de útero aislado de rata que evalúan la contractilidad miometrial inducida por oxitocina, en los cuales se emplean entre 10 y 15 preparaciones tisulares por grupo experimental para detectar cambios significativos en los parámetros de contracción uterina ^20^^,^^21^. Considerando la variabilidad reportada para este modelo y la magnitud del efecto esperada para compuestos con actividad espasmolítica, se estableció un tamaño mínimo de 15 segmentos uterinos por grupo experimental, lo cual se considera adecuado para identificar diferencias estadísticamente significativas bajo condiciones experimentales controladas.

### Sustancias químicas y fármacos

Todas las sales utilizadas para la preparación de la solución de Tyrode y para el tamizaje fitoquímico fueron de grado analítico y adquiridas de Sigma Chemical (St. Louis, MO, EE. UU.). La oxitocina (Pitocin 10 U/mL®, Laboratorios SIEGFRIED, Lima, Perú) y el cipionato de estradiol (Estrogal 2 mg/mL®) se obtuvieron en una farmacia local.

### Recolección del material vegetal y preparación del extracto

*P. galioides* Kunth (conocida comúnmente como congona) fue recolectada en el centro poblado de Chichipe, distrito de Huayllapampa (Áncash, Perú), ubicado a 3132 m s. n. m. Las plantas fueron seleccionadas asegurando ausencia de daño físico, exposición a pesticidas u otros contaminantes. La identificación taxonómica fue realizado por un biólogo certificado del Museo de Historia Natural de la Universidad Nacional Mayor de San Marcos (Constancia 147-USM-MHN-2024).

Las hojas se lavaron con agua destilada, se secaron durante siete días a 40 °C con ventilación continua y se molieron hasta obtener un polvo uniforme. El extracto hidroalcohólico se preparó mediante maceración de 250 g de hojas pulverizadas en 1 L de etanol al 70% durante 7 días con agitación diaria. El extracto se filtró con papel Whatman N.° 3 y el filtrado se evaporó a 40 °C mediante un rotavapor (Buchi®). El extracto concentrado se secó posteriormente a 40 °C durante 7 días y se almacenó a −30 °C.

El rendimiento de extracción fue del 2%, equivalente a 2 g de extracto hidroalcohólico crudo por cada 100 g de material vegetal macerado. Para los ensayos de bioactividad, los extractos se disolvieron en Tween 80 al 10%.

### Tamizaje fitoquímico preliminar

El extracto hidroalcohólico de *P. galioides* fue sometido a un tamizaje cualitativo de metabolitos secundarios mediante métodos fitoquímicos estándar previamente descritos ^22^. Las pruebas incluyeron: reacción de Liebermann-Burchard para esteroles y triterpenoides; prueba de Shinoda para flavonoides; prueba de cloruro férrico para compuestos fenólicos; y pruebas de Dragendorff, Wagner y Mayer para alcaloides.

Adicionalmente, se realizaron la prueba de gelatina para taninos, la reacción de Baljet para saponinas, la reacción de Bortanger para antraquinonas y naftoquinonas, la reacción de Salkowski para terpenoides y flavonoides esteroidales, la prueba de Bertrand para heterósidos cardiotónicos y lactonas sesquiterpénicas, y el ensayo de vainillina/H₂SO₄ para determinar el índice de espuma. Todas las reacciones se efectuaron bajo condiciones controladas de laboratorio, utilizando reactivos de grado analítico y metodologías cualitativas estandarizadas para garantizar la reproducibilidad.

### Animales de experimentación

Se emplearon 15 ratas hembras adultas vírgenes de la cepa Holtzman, de tres meses de edad y peso entre 250-300 g, obtenidas del bioterio de la Universidad Nacional Agraria La Molina (Lima, Perú). Los animales fueron alojados en el bioterio de la Facultad de Medicina de la Universidad Nacional Mayor de San Marcos (UNMSM) bajo condiciones controladas de temperatura (21-25 °C), humedad (50-60%) y ciclo luz/oscuridad de 12 horas, con acceso libre a alimento y agua. Antes del experimento se realizó un periodo de aclimatación de siete días.

Los criterios de inclusión, definidos a priori, incluyeron ratas hembras adultas sanas, vírgenes, dentro del rango de peso establecido. Se excluyeron animales que presentaron signos clínicos de enfermedad, lesiones, pérdida significativa de peso o cualquier condición que pudiera afectar la respuesta fisiológica del tejido uterino. Así también, se descartaron aquellos segmentos uterinos que no presentaron actividad contráctil basal adecuada o que no respondieron al estímulo con oxitocina durante el periodo de estabilización.

Durante el desarrollo del experimento no se excluyeron animales ni unidades experimentales del análisis. Todos los segmentos uterinos obtenidos cumplieron con los criterios de viabilidad establecidos, evidenciando actividad contráctil basal y respuesta adecuada al estímulo con oxitocina durante el periodo de estabilización. Por lo tanto, todos los datos obtenidos fueron incluidos en el análisis final.

### Preparación y montaje del útero aislado

Las ratas fueron pretratadas mediante inyección intramuscular de 0,6 mg de cipionato de estradiol (Estrogal® 2 mg/mL) 48 horas antes del experimento. Los animales fueron anestesiados con pentobarbital sódico (60 mg/kg, i.p.) y sacrificados por dislocación cervical.

Se realizó una laparotomía media para extraer el útero, el cual se limpió de tejido conectivo y se colocó en una placa Petri con solución de Tyrode a 37 °C. Cada cuerno uterino se seccionó en fragmentos de 10 mm y se montó en un baño de órgano de 5 mL con solución de Tyrode oxigenada (95% O₂, 5% CO₂) mantenida a 37 °C.

La solución de Tyrode presentó la siguiente composición (por litro): NaCl 136 mM, KCl 2,68 mM, CaCl₂ 1,80 mM, MgCl₂ 1,05 mM, NaHCO₃ 11,9 mM, NaH₂PO₄ 0,42 mM y glucosa 5,55 mM. Las tiras de tejido se fijaron con hilo quirúrgico y se conectaron a un transductor de fuerza isométrica (ADInstruments, Australia), permitiendo el registro de la actividad contráctil mediante el software LabChart®.

Las preparaciones se estabilizaron durante 60 minutos, con cambios de solución cada 20 minutos, manteniendo una tensión basal de 1 g.

Para minimizar posibles variables de confusión con el fin de asegurar la reproducibilidad de las mediciones de contractilidad uterina, todos los experimentos se realizaron bajo condiciones experimentales estandarizadas de temperatura, oxigenación y tensión basal en el sistema de baño de órganos.

### Ensayos farmacológicos

Tras el periodo de estabilización, se registró la actividad contráctil basal durante tres minutos. Posteriormente, el tejido uterino fue estimulado con 0,2 mL de solución de oxitocina (10⁻⁶ M), registrándose la contracción durante otros tres minutos. Luego, se administraron secuencialmente los extractos hidroalcohólicos (0,1%, 1% y 10%) con intervalos de tres minutos.

Al finalizar el experimento, se realizaron cinco lavados consecutivos y se evaluó la recuperación de la actividad inducida por oxitocina para confirmar la viabilidad del tejido. Los parámetros analizados fueron frecuencia (contracciones/3 minutos), intensidad (g de fuerza) y duración (segundos) de las contracciones uterinas.

Debido a la naturaleza del experimento y al manejo directo de las preparaciones en el sistema de baño de órganos, el investigador que realizó los registros de la actividad contráctil tuvo conocimiento de los tratamientos administrados. Sin embargo, las mediciones de los parámetros de contractilidad uterina (frecuencia, duración e intensidad) fueron obtenidas mediante un sistema automatizado de registro isométrico y posteriormente analizadas mediante software especializado, lo que redujo la posibilidad de sesgo en la evaluación de los resultados y en el análisis de los datos.

### Análisis estadístico

La normalidad de los datos se evaluó mediante la prueba de Shapiro-Wilk, la cual indicó una distribución no normal (p < 0,05). En consecuencia, se utilizó la prueba de rangos con signo de Wilcoxon para comparar los valores antes y después del tratamiento, y la prueba de Kruskal-Wallis para comparaciones entre grupos. Para todos los análisis se utilizó el paquete estadístico IBM SPSS versión 26. Se acepto un valor de p < 0,05 como significativo.

El porcentaje de inhibición para cada parámetro se calculó mediante la siguiente fórmula:



### Aspectos éticos

El estudio fue aprobado por el Comité de Ética de la Facultad de Medicina de la UNMSM (código: 0072-2024), el cual verificó la idoneidad del proyecto, así como garantizó que se brinden los cuidados adecuados implicados durante el alojamiento, los factores ambientales, práctica de sujeción, inyección y eutanasia de las ratas. Todos los procedimientos se realizaron de acuerdo con las directrices para el cuidado y uso de animales de laboratorio y las normas internacionales para investigación preclínica, garantizando un manejo adecuado, sujeción, anestesia y eutanasia ^23^^,^^24^. Asimismo, el estudio se desarrolló en conformidad con los lineamientos establecidos por la declaración ARRIVE ^25^. 

## RESULTADOS

### Perfil fitoquímico cualitativo del extracto hidroalcohólico de *Peperomia galioides* Kunth

El tamizaje fitoquímico cualitativo del extracto hidroalcohólico de *P. galioides* (congona) reveló la presencia de diversos metabolitos secundarios. Se observó una presencia alta (+++) de compuestos fenólicos mediante la prueba de cloruro férrico, así como de glucósidos detectados por la prueba de vainillina/H₂SO₄. La prueba de Shinoda también evidenció una presencia alta (+++) de flavonoides.

Por otro lado, se detectó una presencia moderada (++) de heterósidos cardiotónicos y lactonas sesquiterpénicas mediante la prueba de Baljet. Se observó una presencia baja (+) de antraquinonas y naftoquinonas mediante la prueba de Bortanger, así como de terpenoides y flavonoides con núcleo esteroide mediante la prueba de Salkowski.

No se encontró evidencia (−) de taninos (prueba de gelatina), esteroides y triterpenoides (prueba de Liebermann-Burchard), alcaloides (pruebas de Dragendorff, Wagner, Bertrand y Mayer) ni saponinas (índice de espuma).

### Efecto del extracto hidroalcohólico de *Peperomia galioides* Kunth sobre los parámetros de contracción uterina inducida por oxitocina

El extracto hidroalcohólico de *P. galioides* ejerció efectos moduladores sobre la contractilidad uterina inducida por oxitocina, evaluada mediante la frecuencia, duración e intensidad de las contracciones (figura 1).

**Figura 1:** Efecto del extracto hidroalcohólico de *Peperomia galioides* Kunth sobre la frecuencia, duración e intensidad de las contracciones uterinas inducidas por oxitocina en ratas. Las ratas fueron tratadas con diferentes concentraciones del extracto (0,1%, 1% y 10%) y comparadas con el grupo control (oxitocina sola). Se evaluaron tres parámetros: frecuencia de contracción (A), duración (B) e intensidad (C). Los datos se expresan como media ± desviación estándar.

El análisis estadístico se realizó mediante la prueba no paramétrica de rangos con signo de Wilcoxon para muestras apareadas, ajustada por el método de Benjamini, Krieger y Yekutieli (FDR 1 %). p < 0,05 (*).

En relación con la frecuencia de contracción, todas las concentraciones evaluadas del extracto redujeron significativamente las contracciones inducidas por oxitocina en comparación con el grupo control (p < 0,05).

Respecto a la duración de la contracción, solo las concentraciones al 1% y 10% produjeron una reducción estadísticamente significativa en comparación con la concentración al 0,1% y el grupo control (p < 0,05). No se detectaron diferencias significativas entre las concentraciones al 0,1% y 1%, ni en comparación con el control.

En cuanto a la intensidad de contracción, también se observó una reducción estadísticamente significativa con las concentraciones al 1% y 10% en comparación con el grupo tratado con 0,1% y el grupo control (p < 0,05).

En conjunto, estos hallazgos sugieren un efecto dependiente de la concentración, en el cual las concentraciones al 1% y 10% ejercieron un efecto inhibidor significativo sobre todos los parámetros contráctiles en el modelo de útero aislado de rata.

### Porcentaje de inhibición de los parámetros de contracción uterina

La figura 2 muestra el porcentaje de inhibición respecto a las contracciones inducidas por oxitocina para cada parámetro de contractilidad uterina.

**Figura 2:** Porcentaje de inhibición del extracto hidroalcohólico de Peperomia galioides Kunth sobre los parámetros de contracción uterina inducida por oxitocina. Se calculo el porcentaje de inhibición respecto al grupo control para los tres parámetros evaluados: frecuencia (A), duración (B) e intensidad (C) de la contracción uterina. El extracto se evaluó a concentraciones de 0,1%, 1% y 10%. Los datos se presentan como media ± desviación estándar.

El análisis estadístico se realizó mediante la prueba no paramétrica de Kruskal-Wallis, con corrección FDR al 1 %. p < 0,05 (*), p < 0,01 (**), p < 0,001 (***).

El extracto al 10% presentó el mayor porcentaje de inhibición en los tres parámetros evaluados. Para la frecuencia de contracción, las concentraciones al 0,1%, 1% (p < 0,05) y 10% (p < 0,001) difirieron significativamente del grupo control.

En cuanto a la duración de la contracción, solo la concentración al 10% produjo una inhibición significativamente mayor en comparación con el grupo control (p < 0,05), así como con las concentraciones al 0,1% y 1%. No se observaron diferencias significativas entre las concentraciones al 0,1% y 1%.

Respecto a la intensidad de contracción, únicamente el extracto al 10% mostró una diferencia significativa frente al control (p < 0,001), mientras que las concentraciones al 0,1% y 1% no difirieron significativamente del control ni entre sí.

## DISCUSIÓN

Los resultados demostraron que el extracto de *P. galioides* Kunth inhibe los tres parámetros evaluados de contracción uterina de manera dependiente de la dosis. En particular, la frecuencia de contracción fue significativamente menor que la del grupo control en todas las concentraciones evaluadas, siendo la concentración más alta la más efectiva (p < 0,05). Asimismo, al analizar el porcentaje de inhibición de la intensidad, solo la concentración al 10% mostró una diferencia estadísticamente significativa, lo que indica que a dosis elevadas el extracto posee una clara capacidad para atenuar la fuerza contráctil miometrial inducida por oxitocina.

En relación con la duración e intensidad de las contracciones, aunque se observó una tendencia decreciente en los valores absolutos al aumentar la concentración del extracto, el análisis del porcentaje de inhibición indicó que solo la concentración al 10% redujo significativamente la intensidad de contracción en comparación con el control (p < 0,01). Este efecto respalda la hipótesis de que *P. galioides* podría modular la contractilidad miometrial interfiriendo con los mecanismos fisiológicos que regulan la contracción uterina, particularmente mediante la reducción de la duración de los eventos contráctiles efectivos Se ha descrito que la contractilidad del músculo liso uterino depende en gran medida de la entrada de calcio a través de canales dependientes de voltaje y de los mecanismos intracelulares que regulan la generación de potenciales de acción miometriales, por lo que la inhibición de estas vías puede disminuir la fuerza y duración de la contracción ^26^^,^^27^.

El tamizaje fitoquímico del extracto hidroalcohólico de *P. galioides* Kunth (congona) reveló la presencia de compuestos fenólicos, flavonoides y glucósidos, junto con trazas de otros metabolitos secundarios. Desde el punto de vista farmacológico, estos hallazgos son relevantes, ya que tanto los flavonoides como los compuestos fenólicos han sido ampliamente asociados con actividades antiinflamatorias, antioxidantes y espasmolíticas previamente descritas en especies del género *Peperomia*^28^.

La identificación de flavonoides con evidencia alta (+++) concuerda con estudios previos que destacan su papel en la modulación de la contractilidad del músculo liso y la inhibición de mediadores proinflamatorios, contribuyendo a explicar los potenciales efectos tocolíticos o antiespasmódicos atribuidos tradicionalmente a la planta. Otro aspecto relevante es la actividad estrogénica descrita en otras especies de *Peperomia*, que podría representar un mecanismo adicional. Se ha reportado que compuestos fenólicos presentes en este género poseen afinidad por los receptores de estrógeno, lo que sugiere un potencial para modular vías hormonales relacionadas con la contractilidad uterina ^29^.

Algunos compuestos bioactivos aislados de *Peperomia pellucida* (como derivados del dillapiol y la pelucidina A) han demostrado efectos moduladores sobre los receptores estrogénicos ^29^. En estudios de acoplamiento molecular, la actividad estrogénica pareció estar mediada por un mecanismo clásico dependiente de ligando, como lo sugieren las interacciones de unión entre estos compuestos y los receptores de estrógeno.

Aunque otros metabolitos, como heterósidos cardiotónicos y lactonas sesquiterpénicas, fueron identificados con menor intensidad, su contribución farmacológica potencial no puede descartarse y requiere estudios adicionales para evaluar su papel específico.

Asimismo, estudios previos han demostrado que la pelucidina A es capaz de inhibir la ciclooxigenasa-2 (COX-2) y la óxido nítrico sintasa inducible (iNOS), lo que podría mediar un efecto relajante al reducir la síntesis de prostaglandinas procontráctiles y óxido nítrico en células musculares lisas ^30^^,^^31^. Este mecanismo es compartido con tocolíticos clínicos de primera línea, como los AINE, lo que sugiere una base racional plausible para su potencial uso terapéutico ^5^.

*P. pellucida* también presenta efectos vasorrelajantes mediados por mecanismos^32^ dependientes de óxido nítrico. Estos efectos han sido observados en tejidos vasculares como la aorta, donde se evidencia relajación frente a estímulos contráctiles como fenilefrina y KCl. Dado que el músculo liso uterino comparte similitudes fisiológicas con otros tipos de músculo liso, es posible que *P. pellucida* también ejerza un efecto relajante a nivel uterino, particularmente en contextos donde la regulación del tono muscular es esencial para mantener la quiescencia uterina y controlar el trabajo de parto. Aunque donadores de óxido nítrico como la nitroglicerina no se emplean rutinariamente como tocolíticos, pueden utilizarse en contextos clínicos específicos debido a su efecto relajante sobre el músculo liso uterino ^33^.

La activación de los receptores de estrógeno incrementa la producción endotelial de óxido nítrico, el cual desempeña un papel clave en la vasodilatación uterina durante el embarazo ^34^. Dado que el estrógeno favorece la producción de óxido nítrico, también podría tener un papel central en la regulación del patrón y la frecuencia de las contracciones uterinas durante el trabajo de parto. Por tanto, es plausible que la actividad estrogénica de *P. pellucida* pueda modificar la actividad contráctil.

A pesar de los hallazgos obtenidos, es importante reconocer algunas limitaciones del estudio. En primer lugar, no se realizaron ensayos comparativos con tocolíticos de referencia, lo que limita la posibilidad de establecer comparaciones directas sobre la potencia o el mecanismo farmacológico del extracto evaluado; sin embargo, esta limitación no invalida los resultados, ya que el objetivo principal fue determinar el efecto modulador del extracto sobre la contractilidad uterina en un modelo experimental de útero aislado inducido por oxitocina, ampliamente utilizado y validado para evaluar la actividad uterina. Asimismo, el uso de un extracto hidroalcohólico crudo constituye otra limitación, debido a que no permite identificar ni cuantificar los compuestos bioactivos responsables del efecto observado, lo que podría generar variabilidad en la composición química; no obstante, este posible efecto se minimizó mediante la estandarización del proceso de recolección, identificación, secado y extracción del material vegetal, así como mediante la realización de un tamizaje fitoquímico preliminar que confirmó la presencia de metabolitos secundarios potencialmente activos.

En términos generales, estos resultados respaldan parcialmente el uso tradicional de la congona como planta medicinal y proporcionan una base para futuras investigaciones orientadas a la caracterización y cuantificación de sus compuestos bioactivos, así como a la realización de ensayos comparativos que permitan esclarecer con mayor precisión el mecanismo de acción. En este contexto, futuras investigaciones deberían priorizar ensayos específicos *in vitro* e *in vivo* en tejido uterino para definir el perfil farmacodinámico de esta planta y evaluar su potencial aplicación obstétrica en el parto prematuro. Asimismo, considerando que los hallazgos provienen de un modelo experimental en útero aislado de rata, su extrapolación a otras especies, incluida la humana, debe realizarse con cautela. Por ello, se requieren estudios adicionales que evalúen su eficacia y seguridad antes de considerar cualquier posible aplicación clínica.

En conclusión, los resultados del presente estudio demuestran que el extracto hidroalcohólico de hojas de *Peperomia galioides* Kunth (congona) ejerce un efecto inhibidor significativo sobre la contractilidad uterina inducida por oxitocina en un modelo de útero aislado de rata no gestante, particularmente a la concentración del 10%.
